# Determination of eight artificial sweeteners and common *Stevia rebaudiana* glycosides in non-alcoholic and alcoholic beverages by reversed-phase liquid chromatography coupled with tandem mass spectrometry

**DOI:** 10.1007/s00216-014-8355-x

**Published:** 2014-12-04

**Authors:** Paweł Kubica, Jacek Namieśnik, Andrzej Wasik

**Affiliations:** Department of Analytical Chemistry, Faculty of Chemistry, Gdańsk University of Technology, Narutowicza 11/12, 80-233 Gdańsk, Poland

**Keywords:** Artificial sweeteners, Steviol glycosides, *Stevia rebaudiana*, Tandem mass spectrometry, Liquid chromatography

## Abstract

The method for the determination of acesulfame-K, saccharine, cyclamate, aspartame, sucralose, alitame, neohesperidin dihydrochalcone, neotame and five common steviol glycosides (rebaudioside A, rebaudioside C, steviol, steviolbioside and stevioside) in soft and alcoholic beverages was developed using high-performance liquid chromatography and tandem mass spectrometry with electrospray ionisation (HPLC-ESI-MS/MS). To the best of our knowledge, this is the first work that presents an HPLC-ESI-MS/MS method which allows for the simultaneous determination of all EU-authorised high-potency sweeteners (thaumatin being the only exception) in one analytical run. The minimalistic sample preparation procedure consisted of only two operations; dilution and centrifugation. Linearity, limits of detection and quantitation, repeatability, and trueness of the method were evaluated. The obtained recoveries at three tested concentration levels varied from 97.0 to 105.7 %, with relative standard deviations lower than 4.1 %. The proposed method was successfully applied for the determination of sweeteners in 24 samples of different soft and alcoholic drinks.

**Electronic supplementary material:**

The online version of this article (doi:10.1007/s00216-014-8355-x) contains supplementary material, which is available to authorized users.

## Introduction

Sweetness is probably one of the most appreciated features of the food we eat. However, not all consumers want to (or can) consume sugars—the most obvious source of sweetness. The artificial sweeteners which are commonly used in the food industry seem to be an ideal, non-caloric replacement for sweet-tasting sugars.

These sweeteners and their mixtures play an important role in the modern food industry since they provide a means to fulfil the consumer’s demand for sweet, tooth-friendly, reduced-calorie food. The newest members of the European Union (EU)-authorised sweeteners’ family are steviol glycosides. The leaves of *Stevia* contain mostly stevioside and rebaudioside A. Other glycosides present include rebaudioside C, dulcoside A, steviolbioside, rubusoside and rebaudiosides D, E and F [1, 2]. Rebaudioside A is the most desired component of *Stevia* leaf extracts, due to its highest sweetening potency and the least pronounced bitter aftertaste. Steviol glycosides are the second completely natural EU-authorised sweeteners, thaumatin being the first.

While the demand for sweetness without calories is continuously growing, there are still controversies concerning the safety of high-potency sweeteners. Therefore, to ensure consumer safety and trust, the content of such sweeteners in food is strictly regulated by regional or national legislation [3–7]. Proper control over food manufacturing processes calls for appropriate analytical methods, capable of providing reliable results when analysing food samples, usually characterised by quite a complex matrix.

Among all the available methods for the determination of artificial sweeteners in foodstuffs, reversed-phase high-performance liquid chromatography (RP-HPLC) coupled with a variety of detectors is probably the most popular choice [8]. Nowadays, RP-HPLC coupled with tandem mass spectrometry is becoming more and more popular due to its high selectivity and multianalyte capability [9–14], which is an important feature since different sweeteners are frequently used in mixtures to achieve the desired taste, flavour or texture of an end product. Methods capable of separating and quantifying multiple high-potency sweeteners in foods are known [8, 10, 15–29], but, to the best of our knowledge, their ability to determine *Stevia*-based sweeteners in mixtures with other non-caloric sweeteners was not yet demonstrated in practice.

The purpose of this project was to develop a quick, simple and robust method for the determination of almost all EU-authorised high-potency sweeteners, including steviol glycosides. The only exception is thaumatin which is a protein and cannot be quantified with this method due to the incompatibility with the separation conditions used. According to the proposed method, analytes are separated by RP-HPLC, and later on detected and quantified using tandem mass spectrometry. The sample preparation procedure is limited to the dilution and centrifugation (or filtration) of the samples. The method allows the quantification of 14 compounds in one 16-min-long analytical run. Low values of limits of quantitation (LOQ), high recoveries, and satisfactory repeatability make it suitable for application in food control laboratories, as demonstrated by the analysis of 24 samples of different soft and alcoholic beverages.

## Materials and methods

### Chemicals

Standards of artificial sweeteners and steviol glycosides were obtained from different sources: acesulfame-K from Nutrinova (Frankfurt am Main, Germany); saccharine, sucralose and neohesperidin DC from Sigma-Aldrich (St. Louis, USA); aspartame from Ajinomoto Foods Europe (Nesle, France); cyclamate from Merck KGaA (Darmstadt, Germany); alitame from Frapp’s Pharma (Hong Kong, China); neotame from CHEMOS (Regenstauf, Germany); and rebaudioside A, stevioside, rebaudioside C, dulcoside A, steviolbioside and steviol from LGC Standards (Łomianki, Poland). As the internal standard (IS), sodium *N*-(2-methylcyclohexyl)sulfamate was used [27]. Acetonitrile (ACN), methanol (MeOH) and acetone were purchased from Merck KGaA (Darmstadt, Germany). Acetic acid (AA) was obtained from POCH (Gliwice, Poland). Ultrapure water was prepared using the HLP5 system from Hydrolab (Wiślina, Poland).

### Samples

Twenty-one samples of popular soft and alcoholic drinks and three samples of instant drink powders from different producers were purchased in local shops. Most products were labelled as containing steviol glycosides, although other drinks were also included for method-testing purposes.

### Preparation of standards and calibration solutions

The stock solutions of acesulfame-K, saccharine, neohesperidin DC, aspartame, sucralose, cyclamate, alitame, neotame, rebaudioside A, stevioside, rebaudioside C, dulcoside A, steviolbioside and steviol were prepared by dissolving the appropriate amount of pure standard in the mixture of ACN and H_2_O (60 + 40). The final concentration of each standard was around 50 μg/mL. The calibration solutions were prepared by mixing and diluting the stock solutions with the mobile-phase component A to obtain 5, 20, 50, 100, 200, 400 and 800 ng/mL of acesulfame-K, saccharin, neohesperidin DC, aspartame, sucralose, cyclamate, alitame and neotame, while concentrations of rebaudioside A, stevioside, rebaudioside C, dulcoside A, steviolbioside and steviol were 5, 20, 100, 300, 600, 1000 and 1600 ng/mL, respectively. The IS concentration was kept at 50 ng/mL in each calibration solution. All solutions were stored in a refrigerator at 4 °C; new solutions were prepared monthly.

### Sample preparation and fortification procedures

Before sample preparation, all samples of soft and alcoholic drinks were degassed by sonication for 15 min. Instant drinks were prepared according to the manufacturer’s directions. Samples were diluted with mobile-phase component A in order to fall within calibration curve concentration range. In practice, a hundred times of dilution was appropriate, i.e. 100 μL of each sample and 50 μL of IS solution were placed in a 10-mL volumetric flask and filled up to the mark. Approximately 1.5 mL of this solution was placed in an Eppendorf tube and centrifuged for 5 min at 7000 rpm. Supernatant was collected, placed in the autosampler vial and analysed.

Fortified samples (three concentration levels) were prepared using the Sprite™ drink (old recipe, free from steviol glycosides and other high-potency sweeteners) as a matrix. Sweeteners were dissolved in Sprite™ to get a concentration of 500 μg/mL each. This mixture was later on diluted with Sprite™ to obtain the concentration levels of 10, 25 and 60 μg/mL. Fortified samples were used for repeatability and apparent recovery estimation.

### MS/MS conditions

All analyses were done using a Shimadzu LCMS-8050 triple quadrupole mass spectrometer (Shimadzu, Japan) equipped with an ESI source working in the polarity-switching MRM mode. Positive detection mode for aspartame, alitame and neotame was selected to increase the sensitivity. Data acquisition and analysis were accomplished with LabSolutions 5.60 SP1 software. The specific MRM transitions were chosen in the flow injection mode. The optimum detection conditions are presented in Table S1 in the Electronic Supplementary Material (ESM).

### HPLC conditions

**Fig. 1:**
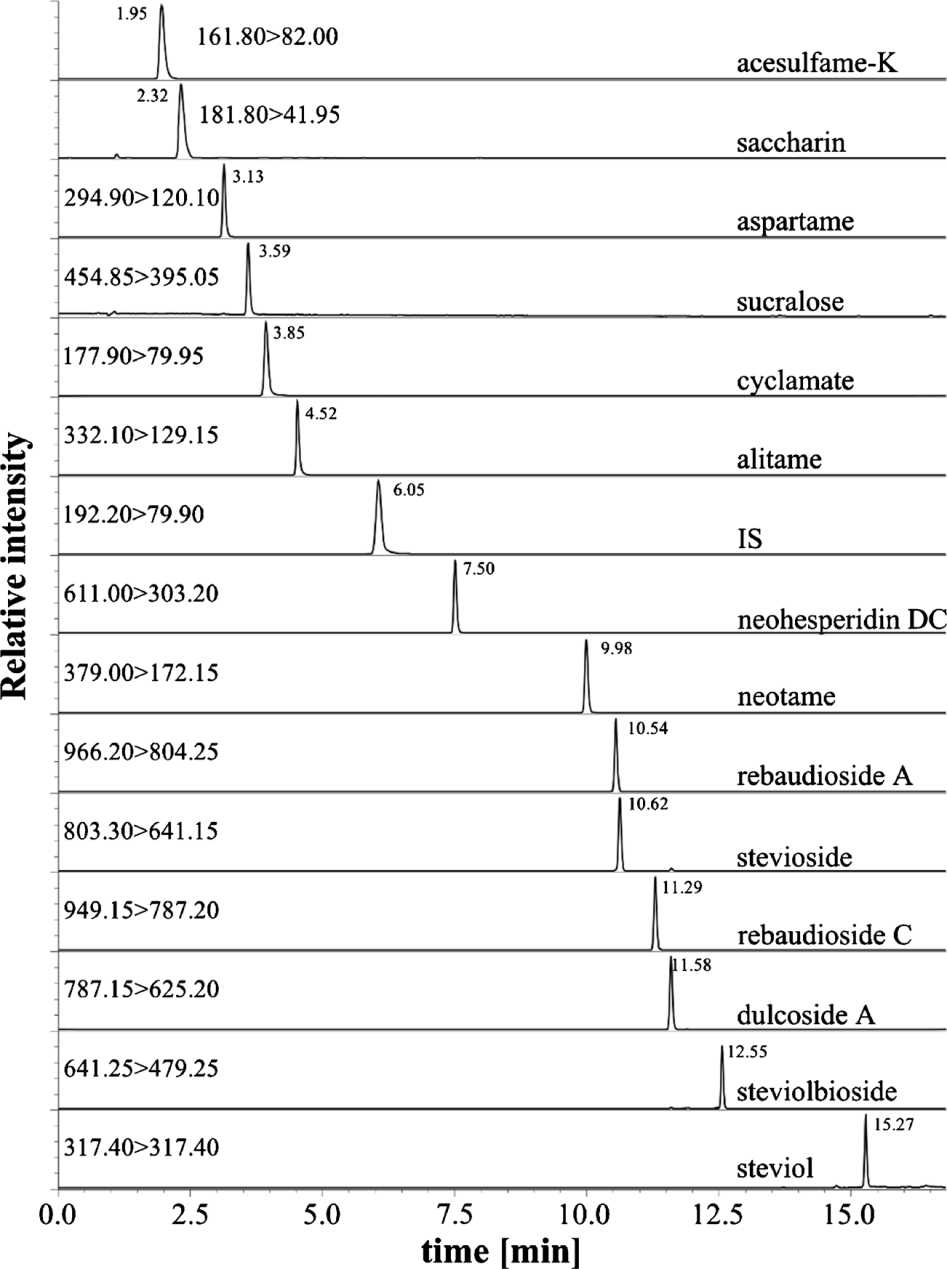
Example of a chromatogram of standard mixture (200 ng/mL each)—see conditions in the text

The chromatographic separation was carried out using an HPLC system (Shimadzu, Japan) consisting of a degasser DGU-20A5R, controller CBM-20A, binary pump Nexera X2 LC-30 AD, autosampler Nexera X2 SIL-30AC and column oven CTO-20AC. The analytes were separated on an Ascentis Express C18 column (Supelco, Belefonte, PA, 100 mm × 4.6 mm, 2.7 μm). The temperature of the column oven was set to 40 °C, the flow rate was kept at 0.8 mL/min and the injection volume was set to 2 μL. The mobile phase used for the separation was H_2_O + MeOH + acetone (75 + 20 + 5) with 0.1 % *v*/*v* of AA (component A) and ACN + acetone (95 + 5) with 0.1 % *v*/*v* of AA (component B). The chromatographic separation was performed in gradient elution mode: 0 min (0 % B), 10 min (30 % B), 15 min (70 % B) and 16 min (70 % B). The total time of the chromatographic run was 16 min, while the column equilibration time was set to 8 min. The chromatogram presenting the separation of analytes is shown in Fig. 1.

## Results and discussion

### Separation and detection of analytes

In case of sucralose, acetic acid adduct was chosen (454.85 *m*/*z*) as the parent ion and 395.05 as the fragment ion. The choice was dictated by the fact that the intensity of this transition (454.85 → 395.05) was higher than the transition of the pseudomolecular ion of sucralose (395.05) to its fragments: 359.15 or 87.05. The fragmentation of steviol molecule was not observed neither in negative nor in positive mode. The higher intensity of the pseudomolecular ion was higher in the negative mode. The absence of fragment ions forced the choice of pseudotransition of steviol 317.40 → 317.40. The best response for aspartame, alitame and neotame was observed in the positive mode of detection.

The addition of a small amount of acetone to both components of the mobile phase resulted in narrower peaks for acesulfame-K, saccharin, aspartame, sucralose, cyclamate, alitame, neohesperidin DC and neotame. The change of peak shapes for rebaudioside A, stevioside, rebaudioside C, dulcoside, steviolbioside and steviol was not observed regardless of acetone addition. Use of two organic components in mobile phase (ACN and MeOH) resulted in better separation of analytes in comparison to the separation achieved with only one organic component. Methanol-only mobile phase had not enough eluting strength to achieve separation in less than 25 min; an incomplete separation of rebaudioside A and stevioside was observed as well. On the other hand, acetonitrile-only mobile phase had higher elution power, but an incomplete separation of acesulfame-K and saccharin, sucralose and cyclamate, and rebaudioside A and stevioside was noticed. The combination of methanol in mobile-phase component A and acetonitrile in mobile-phase component B resulted in a complete separation of these compounds, relatively short analysis time and better separation of rebaudioside A and stevioside. A complete separation was achieved for most of the compounds, except reabudioside A and stevioside (*R*_s_ = 1.2). This was caused by fact that these two compounds differ only by one extra glucose molecule in the structure of RA. However, complete separation is not necessary in that case, since two specific transitions for RA and SV, respectively, can be measured independently.

### Within-laboratory validation

#### Calibration

Seven-point calibration curves were constructed by plotting the ratio of the analyte’s peak area to the peak area of the IS versus the analyte’s concentration (*n* = 3). Different concentration ranges were used for two different classes of sweeteners: 5–800 ng/mL for the artificial ones and 5–1600 ng/mL for steviol glycosides. Calibration curves were linear in the studied concentration range with correlation coefficients of over 0.9987. The weighing factor of 1/*x* was applied to all calibration curves in order to increase the accuracy in the lower concentration range. The values of limit of detection (LOD) were calculated using the following formula: LOD = 3.3 · *S*_*b*_/*a*, where *S*_*b*_ is the standard deviation of the intercept and *a* is the slope of the calibration curve. The values of limit of quantitation (LOQ) were calculated as three times LOD. Quantification limits were between 3.23 and 13.56 ng/mL, which correspond to the range of 0.323 and 1.36 mg/L in the original sample, assuming a hundred times of dilution of the sample. These values are well below the regulatory limits for all compounds under the study. The values of calibration parameters are presented in Table 1.

**Table 1:** Quantification and validation data for artificial sweeteners and steviol glycosides

Analyte	Calibration curve equation (7 points, *n* = 3)	*S*_*a*_	*S*_*b*_	*r*	LOD [ng/mL]	LOQ [ng/mL]
Acesulfame-K	*y* = 0.04286*x* + 0.099	0.00079	0.059	0.9987	4.52	13.56
Saccharin	*y* = 0.004583*x* + 0.0025	0.000025	0.0018	0.9997	1.32	3.95
Aspartame	*y* = 0.04189*x* − 0.070	0.00028	0.021	0.9996	1.63	4.90
Sucralose	*y* = 0.010964*x* − 0.0158	0.000062	0.0046	0.9997	1.38	4.14
Cyclamate	*y* = 0.02994*x* − 0.0454	0.00013	0.0098	0.9998	1.08	3.23
Alitame	*y* = 0.02816*x* − 0.024	0.00021	0.015	0.9994	1.78	5.35
Neohesperidin DC	*y* = 0.04840*x* − 0.124	0.00045	0.033	0.9991	2.28	6.84
Neotame	*y* = 0.004872*x* − 0.0012	0.000020	0.0029	0.9998	1.98	5.95
Rebaudioside A	*y* = 0.004625*x* − 0.0005	0.000019	0.0029	0.9998	2.04	6.11
Stevioside	*y* = 0.016005*x* − 0.011	0.000071	0.010	0.9998	2.16	6.48
Rebaudioside C	*y* = 0.007403*x* − 0.0168	0.000035	0.0052	0.9997	2.33	7.00
Dulcoside A	*y* = 0.002522*x* + 0.0022	0.000014	0.0021	0.9997	2.69	8.08
Steviolbioside	*y* = 0.05032*x* + 0.131	0.00028	0.042	0.9997	2.74	8.23
Steviol	*y* = 0.011370*x* − 0.0362	0.000095	0.0070	0.9993	2.04	6.12

*S*_*a*_ standard deviation of the slope, *S*_*b*_ standard deviation of the intercept, *r* correlation coefficient, *LOD* limit of detection, *LOQ* limit of quantitation, *n* number of measurements

#### Trueness and repeatability

The trueness of the results was assessed in terms of apparent recoveries using spiked Sprite™ drink as a matrix. Samples spiked at three concentration levels (10, 25 and 60 μg/mL) were analysed on the same day (six replicates of each concentration level). Recoveries varied between 97.0 and 105.7 %, while the relative standard deviations (%RSD) of the results were in the range of 0.4–4.1 %. The recovery data and %RSD values indicate good method accuracy and precision. No matrix effects were observed, thanks to the use of an internal standard, significant dilution of the samples and complete separation of analytes.

Repeatability, expressed as between-day precision during the next three consecutive days, was estimated by analysing a set of samples (*n* = 6) spiked at one concentration level (600 ng/mL after sample preparation step). The %RSD of the results were in the range of 1.1–4.5 %, very close to the within-day precision. This demonstrates that the method provides consistent, day-by-day results. Detailed data concerning trueness and repeatability are presented in ESM Table S2.

### Analysis of real samples

Samples were bought in local shops, and attention was paid to ensure their diversity. Three types of drinks were analysed: non-carbonated and carbonated soft drinks, and carbonated alcoholic beverages (beers). Most of the samples (18) were labelled as containing steviol glycosides, though beverages sweetened with other compounds were also taken into account. According to the regulations [5, 6], the content of steviol glycosides should be expressed as the sum of steviol equivalents. The equivalents of steviol are calculated for each glycoside separately using the following multiplication factors: steviol (1.000), stevioside (0.395), rebaudioside A (0.329), rebaudioside C (0.334), dulcoside A (0.400) and steviolbioside (0.496). For the majority of tested samples, the sum of steviol equivalents was within the acceptable limit (60 mg/L)—see Table 2 for details. In two cases (NCNA4 and NCNA8), the legal limit was exceeded.

**Table 2:** Concentrations of artificial sweeteners and steviol glycosides in soft and alcoholic drinks: analysis of real samples. Only detected compounds are shown

Sample type	Name of sample	Acesulfame-K	Saccharin	Aspartame	Cyclamate	Neohesperidin DC	Rebaudioside A^a^	Stevioside^a^	Rebaudioside C^a^	Steviolbioside^a^
		350	80	600	250	30	60^b^			
MUD^c^ in soft drinks [mg/L]										
Detected sweetener content [mg/L] ± SD (*n* = 3)										
Carbonated non-alcoholic	CNA1					0.999 ± 0.052	34.30 ± 0.28	0.616 ± 0.019		
	CNA2	None detected, in accordance with product label								
	CNA3	None detected, in accordance with product label								
	CNA4					1.071 ± 0.071				
	CNA5						17.02 ± 0.36	10.62 ± 0.22	2.542 ± 0.080	
Non-carbonated non-alcoholic	NCNA1						12.64 ± 0.24			
	NCNA2						17.08 ± 0.22	0.310 ± 0.012		
	NCNA3						10.672 ± 0.059	7.70 ± 0.24	1.714 ± 0.019	
	NCNA4					3.02 ± 0.16	53.69 ± 0.99	12.25 ± 0.43	2.498 ± 0.047	
	NCNA5						9.42 ± 0.24	6.01 ± 0.15	1.479 ± 0.018	
	NCNA6						16.48 ± 0.36	6.85 ± 0.25	0.8595 ± 0.0064	0.434 ± 0.018
	NCNA7						11.12 ± 0.43			
	NCNA8						47.94 ± 0.26	28.5 ± 1.1	1.667 ± 0.025	0.784 ± 0.069
	NCNA9						21.91 ± 0.2	13.96 ± 0.21	0.6981 ± 0.0077	
	NCNA10						35.4 ± 1.3	0.691 ± 0.037		
	NCNA11						51.65 ± 0.13	0.7726 ± 0.0071		
MUD in alcoholic drinks [mg/L]										
Carbonated alcoholic	CA1						18.29 ± 0.59	0.475 ± 0.015		
	CA2						16.20 ± 0.13	0.419 ± 0.028		
	CA3	None detected, in accordance with product label								
	CA4	9.25 ± 0.43	5.95 ± 0.34	9.88 ± 0.17	35.0 ± 1.1					
	CA5	23.39 ± 0.19		65.56 ± 0.60						
Detected sweetener content [mg/g] ± SD (*n* = 3)										
Instant drink powders	IDP1						2.131 ± 0.049			
	IDP2						2.19 ± 0.033			
	IDP3						2.802 ± 0.049	1.159 ± 0.055	0.408 ± 0.037	

^a^Expressed as steviol equivalent

^b^The MUD expressed as the sum of steviol equivalents according to commission regulation (EU) no. 1131/2011 of 11 November 2011

^c^Maximum usable dose as set by the Directive 94/35/EC of European Parliament and of the Council of 30 June 1994 and commission regulation (EU) no. 1131/2011 of 11 November 2011

*Stevia-*based sweeteners used in the food industry differ in terms of their composition. Four out of 18 samples were sweetened with highly purified rebaudioside A, and 6 contained rebaudioside A and stevioside. Also, in six cases, three steviol glycosides (rebaudioside A, stevioside, rebaudioside C) were found, and two samples contained four glycosides (rebaudioside A, stevioside, rebaudioside C and steviolbioside). The major compound found in the samples containing a mixture of steviol glycosides was rebaudioside A, the rest being stevioside and, in some cases, minor amounts of rebaudioside C and steviolbioside.

Beverages sweetened with steviol glycosides were found free from other high-potency sweeteners. However, in three cases (CNA1, CNA4 and NCNA4), small amounts of neohesperidin DC were detected. The labels of these beverages did not mention any sweetener other than steviol glycosides, but since neohesperidin DC at low concentrations (up to 3 mg/kg) may be used as a flavour enhancer [7], the composition of these samples is in accordance with the law.

Two out of the five analysed beer samples (CA4 and CA5) were sweetened with artificial high-potency sweeteners. Mixtures of two and four compounds were detected in these cases.

**Fig. 2:**
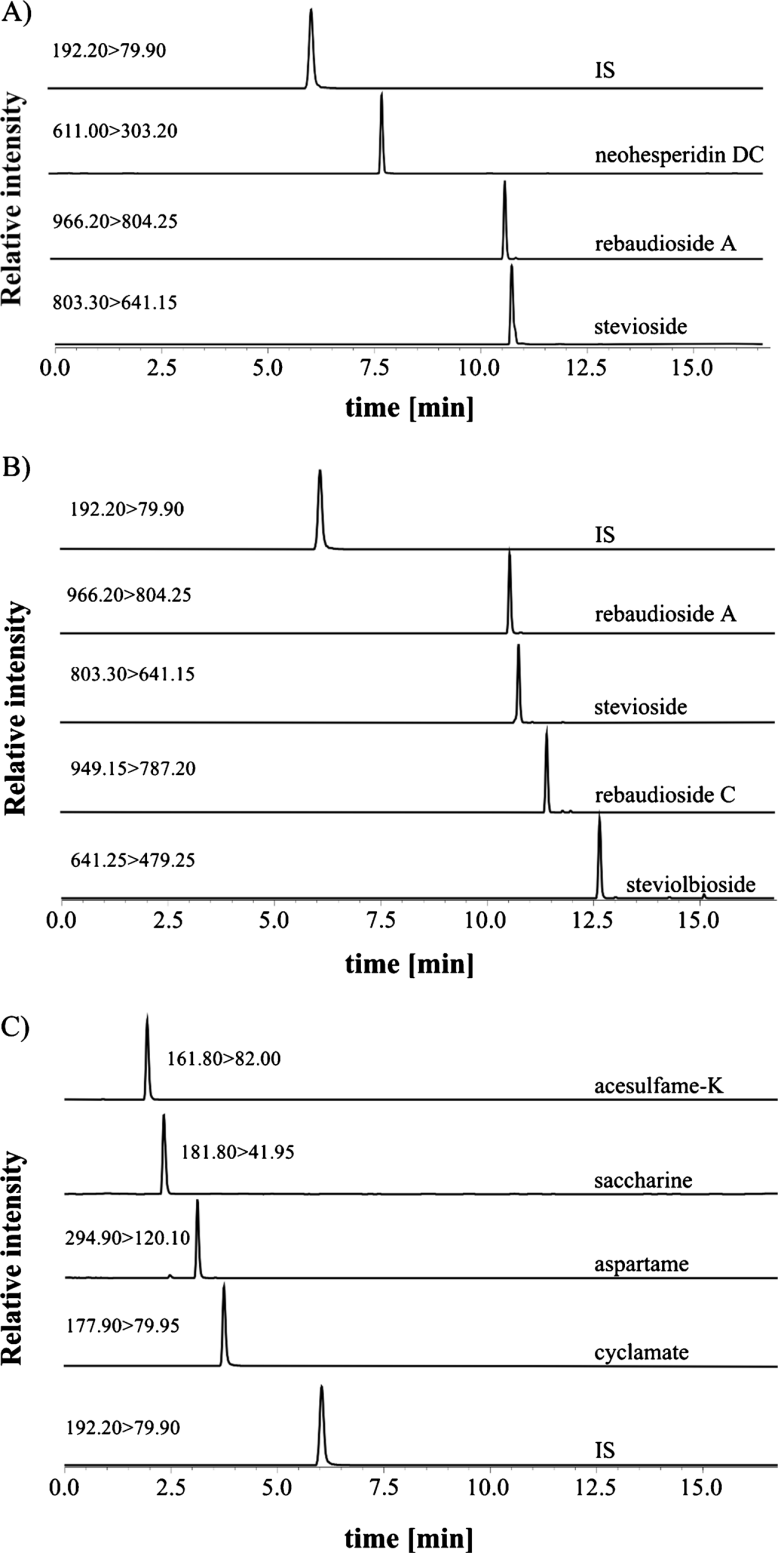
Examples of chromatograms obtained for real samples. *From the top*: **A** sample of CNA1, **B** sample of NCNA6 and **C** sample of CA4

Detailed results of the analysis of all samples are presented in Table 2, and examples of real chromatograms are presented in Fig. 2.

## Conclusions

The presented method allows for the quick determination of all but one EU-authorised high-potency sweeteners in one analytical run. The sample preparation step was simplified to an absolute minimum. It consisted of only two operations: the dilution and centrifugation (or filtration) of the samples. Thanks to the complete separation of analytes and considerable dilution of the analysed samples, no matrix effects were observed. Since the method allows the separation and quantifying of common steviol glycosides, being the components of commercially available *Stevia*-based sweeteners, it can be used to determine their purity/quality. Low limits of quantification, high recoveries and good repeatability of results make it suitable for food quality and safety control. The method was successfully applied for the analysis of sweeteners in alcoholic and non-alcoholic beverages. To the best of our knowledge, this is the first method which allows for the comprehensive analysis of beverages with regard to high-potency sweetener content, including the recently introduced steviol glycosides.

## Electronic supplementary material

Below is the link to the electronic supplementary material.

ESM 1 (PDF 36 kb)
