# Supplementary material for: Determination of eight artificial sweeteners and common Stevia rebaudiana glycosides in non-alcoholic and alcoholic beverages by reversed-phase liquid chromatography coupled with tandem mass spectrometry
Source: Anal Bioanal Chem. 2014 Dec 4;407(5):1505–12. doi: 10.1007/s00216-014-8355-x (PMC4318983; doi:10.1007/s00216-014-8355-x)
Supplement: Supplementary file 1 — (PDF 36 kb) [file 216_2014_8355_MOESM1_ESM.pdf]

Analytical and Bioanalytical Chemistry

Electronic Supplementary Material

Determination of eight artificial sweeteners and common S*tevia rebaudiana* glycosides in non-alcoholic and alcoholic beverages by reversed phase liquid chromatography coupled with tandem mass spectrometry

Paweł Kubica, Jacek Namieśnik, Andrzej Wasik

Table S1 Monitored ion transitions and optimal MS/MS operational parameters

Name	Polarity	Transition	Q1 Pre bias [V]	Collision Energy [V]	Q3 Pre bias [V]
acesuflame-K	-	161.80→82.00	18	16	30
saccharin	-	181.80→41.95	19	26	14
aspartame	+	294.90→120.10	-30	-25	-24
sucralose	-	454.85→395.05*	22	12	26
cyclamate	-	177.90→79.95	19	27	29
alitame	+	332.10→129.15	-16	-18	-27
IS	-	192.20→79.90	14	29	29
neohesperidin DC	-	611.00→303.20	30	37	30
neotame	+	379.00→172.15	-27	-22	-19
rebaudioside A	-	966.20→804.25	38	28	38
stevioside	-	803.30→641.15	30	28	30
rebaudioside C	-	949.15→787.20	38	33	38
dulcoside A	-	787.15→625.20	32	22	30
steviolbioside	-	641.25→479.25	24	42	22
steviol	-	317.40→317.40**	24	15	30

*adduct with acetic acid

** compound does not fragment

**MS/MS operational parameters**					
Nebulizing Gas Flow [L/min]	Heating Gas Flow [L/min]	Interface Temperature [˚C]	DL Temperature [˚C]	Heat Block Temperature [˚C]	Drying Gas Flow [L/min]
3	10	350	250	500	10

DL - Desolvation Line

Table S2 Within- and between-day recoveries (%) and relative standard deviations (RSDs) (%) obtained after HPLC-MS/MS analysis of fortified samples

	Within-day recoveries (RSD, n=6) [%] at different spiking levels			Between-day recoveries (RSD, n=6) [%] at one spiking level (600 ng/mL)		
Analyte	100 ng/mL	250 ng/mL	600 ng/mL	Day 1	Day 2	Day 3
acesulfame-K	100.4 (2.5)	103.5 (2.3)	102.2 (0.6)	103.9 (1.5)	103.5 (1.9)	104.0 (2.2)
saccharin	100.2 (1.9)	97.0 (1.9)	99.0 (1.8)	103.0 (2.9)	103.9 (3.5)	105.2 (2.4)
aspartame	103.3 (2.7)	100.3 (1.1)	102.0 (0.4)	98.4 (1.8)	102.2 (1.2)	101.8 (2.1)
sucralose	104.4 (2.1)	103.0 (1.9)	100.5 (0.4)	103.6 (1.9)	99.6 (2.5)	98.5 (2.6)
cyclamate	101.4 (1.7)	98.1 (2.0)	98.9 (1.1)	97.6 (1.3)	98.9 (1.2)	100.0 (1.5)
alitame	103.8 (2.4)	101.3 (1.4)	101.7 (2.9)	97.8 (2.5)	98.7 (2.1)	97.9 (1.5)
neohesperidin DC	101.9 (3.3)	100.3 (2.3)	98.4 (1.3)	98.8 (1.1)	100.0 (2.3)	101.6 (2.9)
neotame	102.4 (1.8)	99.5 (1.6)	102.1 (1.9)	97.6 (1.2)	97.0 (1.5)	98.1 (1.5)
rebaudioside A	98.4 (2.1)	101.4 (2.9)	103.1 (1.4)	103.3 (2.3)	103.0 (1.8)	101.6 (2.3)
stevioside	102.9 (1.3)	102.4 (3.1)	101.5 (1.7)	102.1 (2.5)	98.3 (1.3)	99.4 (2.7)
rebaudioside C	103.1 (3.4)	102.0 (3.1)	101.9 (2.2)	103.6 (3.0)	102.4 (1.3)	102.1 (2.1)
dulcoside A	105.7 (3.6)	102.6 (2.4)	102.9 (1.4)	105.5 (4.5)	103.2 (1.4)	104.0 (1.8)
steviolbioside	98.0 (4.1)	100.3 (1.8)	102.7 (3.4)	101.9 (2.5)	98.9 (3.6)	98.2 (4.1)
steviol	103.6 (2.8)	100.7 (1.7)	102.1 (2.5)	104.6 (2.9)	98.6 (1.3)	100.8 (2.9)
